# Immunophenotypic insights into diabetes heterogeneity: The role of CD25-expressing T cells in Ghanaian type 1 diabetes cohorts

**DOI:** 10.1016/j.jtauto.2026.100400

**Published:** 2026-09-01

**Authors:** Wilfred Aniagyei, Sophie L. Womelsdorf, Sarah Bittner, Sumaya Mohayideen, Osei Sarfo-Kantanka, Ernest Adankwah, Dorcas O. Owusu, Hillary Y.B. Nafu, Monika M. Vivekanandan, Augustine Yeboah, Joseph F. Arthur, Hubert S. Ahor, Vera Balz, Shadrack Osei Asibey, Agnes Owusu Boateng, Elisabeth Owusu, Ertan Mayatepek, Marc Jacobsen, Richard O. Phillips, Julia Seyfarth

**Affiliations:** a Department of General Pediatrics, Neonatology and Pediatric Cardiology, Medical Faculty and University Hospital Duesseldorf, Heinrich Heine University Duesseldorf, Duesseldorf, Germany; b Kumasi Centre for Collaborative Research in Tropical Medicine (KCCR), Kumasi, Ghana; c Komfo Anokye Teaching Hospital, Kumasi, Ghana; d School of Medicine and Dentistry, College of Health Sciences, Kwame Nkrumah University of Science and Technology (KNUST), Kumasi, Ghana; e Institute for Transplantation Diagnostics and Cell Therapeutics, Medical Faculty, University Hospital, Duesseldorf, Germany

**Keywords:** Type 1 diabetes, Autoimmunity, Inflammation, Atypical diabetes, T cell phenotype

## Abstract

Type 1 diabetes (T1D) in sub-Saharan Africa exhibits heterogeneity, characterised by alternative phenotypes that retain C-peptide levels. The T-cell pathology linked to these atypical phenotypes remains largely unexplored. CD25, the interleukin-2 receptor alpha chain, and IL2RA genetic variants are key T-cell factors implicated in T1D immunopathology and may contribute to the heterogeneity of T1D in an African context.

This study examined IL2RA genotypes and T-cell phenotypes, both ex vivo and following in vitro stimulation, in Ghanaian individuals diagnosed with T1D and classified into groups of low, mid and high C-peptide levels, alongside healthy controls.

Immunophenotyping showed increased CD25 expression on CD4 and CD8 T cells in the diabetes cohorts, correlating with age, C-peptide and HbA1c. IL2RA genotyping revealed limited variability and no link to CD25 expression. An expansion of CD25/CD127 double-positive conventional T cells, mainly in naïve and central memory subsets, was observed in T1D with mid and high C-peptide. Following age-matching, the low C-peptide group also demonstrated an increase in CD25/CD127 double-positive T cells, both ex vivo and after in vitro stimulation.

The findings indicate a common pattern of immune dysregulation characterized by the expansion of activated conventional T cells in T1D with varying C-peptide levels in Ghana.

## Graphical abstract

**Figure:**
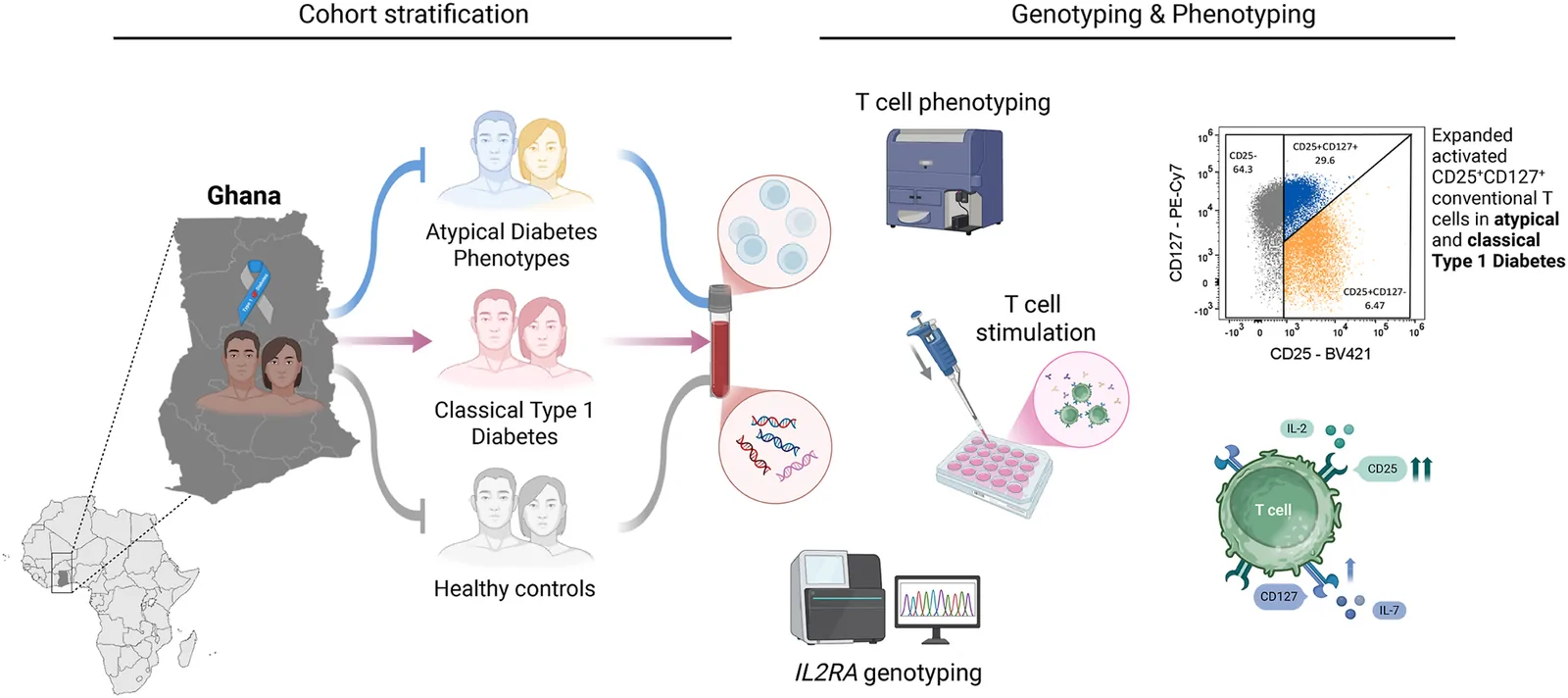
Created in Biorender. Seyfarth, J. (2026) https://BioRender.com/pizl63i.

## 1 Introduction

Type 1 diabetes (T1D) in sub-Saharan Africa is characterised by clinical heterogeneity and diagnostic uncertainty [1]. Recently, we found striking heterogeneity in C-peptide levels in a deeply phenotyped case-control cohort from Ghana comprising insulin-treated long-term T1D and age- and sex-matched healthy controls [2]. Only approximately one-third of clinically diagnosed T1D cases had low C-peptide levels and a phenotype typical of autoimmune T1D, including early onset, lower BMI, HLA class II risk haplotypes, and higher GAD and ZnT8 autoantibodies. Conversely, two-thirds with mid-range or high C-peptide levels resembled healthy controls in HLA class II genes and autoantibodies, indicating a significant presence of atypical diabetes phenotypes such as ketosis-prone diabetes in this group [2]. Therefore, these cohorts provide an exceptional opportunity to investigate the immunological mechanisms underlying diabetes heterogeneity in an African setting. This is of great importance since immune profiling in sub-Saharan African populations is scarce, representing a critical population-level gap in immunologic T1D research.

Central to T cell–mediated immune regulation is Interleukin-2 (IL-2) and its high-affinity receptor, composed of the IL-2 receptor α-chain (CD25), IL-2Rβ and the common γ-chain [3]. CD25 is constitutively expressed at high levels on regulatory T cells (Tregs), where IL-2 signalling is essential for Treg development, homeostasis, and suppressive function [4]. Genetic variation at the IL2RA locus (encoding CD25) is a well-established risk factor for T1D and other autoimmune diseases [5]. Mechanistic studies in humans and NOD mice have shown that IL2RA risk haplotypes or reduced IL-2R signalling impair Treg stability, lower FOXP3 expression, and diminish suppressive capacity, thereby favouring the expansion of pathogenic effector T cells and accelerating T1D development [6,7]. Importantly, CD25 expression is not restricted to Tregs but is also rapidly upregulated on conventional CD4^+^ and CD8^+^ T cells upon activation, where it tunes IL-2 sensitivity, which is crucial for clonal expansion, differentiation, and survival [[8], [9], [10]]. Further dissection of the role of CD25 expression on conventional T cells may reveal novel mechanisms through which CD25 contributes to diabetes pathogenesis. In type 2 diabetes, chronic inflammation is linked to reduced CD4^+^CD25^+^FoxP3^+^ regulatory T cells, impaired IL-2 signalling, and pathogenic effector T cells in insulin resistance [[11], [12], [13]]. Moreover, increased inflammatory markers are frequently observed across various diabetes types, and their expression levels can serve as indicators to differentiate among diabetes subtypes [14]. Therefore, investigating CD25 expression across various T-cell subsets in individuals with varying C-peptide levels may facilitate the characterization of immune dysregulation and activation differences, thereby enhancing the understanding of the underlying immune pathology. This is particularly relevant in the context of immunomodulatory therapies targeting CD25, which are currently under development and being tested for T1D and other autoimmune disorders [15].

In this study, we conducted extensive phenotyping of T cells within previously characterised cohorts of individuals diagnosed with T1D and low, mid and high C-peptide levels, alongside healthy controls [2]. Phenotyping was performed both directly ex vivo and following in vitro activation, with particular emphasis on CD25 expression across regulatory and conventional T cell subsets. Correlations with clinical parameters and the IL2RA genotype were evaluated to identify differences or similarities among the cohorts.

## 2 Materials and methods

### 2.1 Study population

A cross-sectional recruitment of individuals diagnosed with T1D (n = 225) and healthy controls (n = 255) was conducted between August 2021 and December 2023 at the Diabetes Clinic of the Komfo Anokye Teaching Hospital in Kumasi (KATH), Ghana. This study used the same group as previously described (n = 266 per group) [2]. However, the adjusted sample size only included individuals with successful flow cytometry data collection. T1D was clinically diagnosed and classified according to the American Diabetes Association (ADA) criteria, which require persistent insulin dependence and characteristic clinical symptoms, such as polyuria, polydipsia, and ketonuria. The control group comprised individuals with a negative history of autoimmune diseases or diabetes. Study group characteristics are provided in Table 1. Written informed consent was obtained from all study participants (or their legal guardians for participants under 18 years of age). All methods were performed in accordance with the relevant guidelines and regulations. The study was approved by the Ethics Committee Board (KATH IRB/AP/081/20) at KATH.

**Table 1:** Study participant characteristics.

	Control	Diabetes subgroups			*p-value*
		HI	MID	LOW	
Number, *n*	255	78	79	68	
Female/male (*% female*)	175/80 (69%)	57/21 (73%)	58/21 (73%)	37/31 (54%)	0.05
Age [years] (*IQR*)	32 (18-50)	49 (25-57)	31 (20-51)	23 (14-31)	<0.001
Onset Age [years] (*IQR*)	n/a	37 (19-49)	21 (14-36)	13 (9-22)	<0.001
BMI classification [number] (%)					
*Underweight*	33	5	9	20	
*Normal*	107	22	32	38	
*overweight*	70	30	25	5	
*Obese*	45	21	13	5	
BMI (kg/m^2^)	24.2 (20.6-28.4)	26.7 (23.8-30.7)	24.9 (21.5-27.7)	20.2 (18.2-22.9)	<0.001
HbA1c [mmol/mol] (*IQR*)	n/a	74.4 (56.6-93.3)	89.7 (67.6-110)	84.3 (65-102)	0.011
HbA1c [%] (*IQR*)	n/a	9 (7.4-10.9)	9.9 (8.3-11.9)	10.3 (8.1-12.3)	0.008
Insulin dosage/kg [IU per day/kg]	n/a	0.6 (0.4-0.8)	0.6 (0.4-0.9)	0.8 (0.6-1.2)	0.001
Duration of disease [years] (*IQR*)	n/a	7 (3-12)	8 (4-13)	8 (5-11)	0.475

BMI: body mass index; HbA1c: glycated hemoglobin A1c; IQR: interquartile range; IU: international unit; n/a: not applicable.

Data are presented as absolute numbers and percentages for categorical variables, or as medians with interquartile ranges (IQR) for continuous variables. The p-values denote the statistical significance of differences across the four primary study groups (Control, HI, MID, and LOW). Continuous variables were compared using either a one-way ANOVA for normally distributed data, or a Kruskal-Wallis test for non-normally distributed data. Categorical variables were evaluated using appropriate standard tests (e.g., chi-square).

### 2.2 Clinical and biochemical data

The clinical overview and biochemical characteristics of this cohort have been previously described in detail [2]. Random C-peptide levels in the serum were measured using a Roche Cobas 8000 e801 analyser with an electrochemiluminescence immunoassay (ECLIA). Based on these measurements, individuals diagnosed with T1D were stratified into three groups using the following thresholds: LOW (<0.2 nmol/L), MID (0.2-0.6 nmol/L), and HI (>0.6 nmol/L) C-peptide groups, as previously defined [2].

### 2.3 Genotyping

Genomic DNA was extracted from whole blood samples using the QIAamp DNA Mini Kit (Qiagen, Hilden, Germany) according to the manufacturer's instructions. Genotyping for IL2RA was performed using the amplicon-based Next-Generation Sequencing (NGS) methodology previously detailed for IL7RA genotyping [16]. The following SNPs were evaluated: rs11594656, rs12722495, rs1570538, rs35285258, rs41295061, rs2104286, and rs61839660.

### 2.4 Phenotyping of immune cells

Peripheral blood mononuclear cells (PBMCs) were isolated by density gradient centrifugation (Biocoll, Germany) within 1 h of blood collection. For CD25 surface expression, cells were stained with the following antibodies: CD3 (AF700, SK7, BioLegend), CD4 (BV510, RPA-T4, BioLegend), CD8 (FITC, HIT8a, BioLegend), CD127 (PE-Cy7, AO19D5, BioLegend), CD25 (BV421, BC96, BioLegend), CD45RA (APC, HI100, BioLegend), CCR7 (PE, GO43H7, BioLegend), CD27 (PerCP-Cy5.5, 5C3, BioLegend), CD57 (PE-Dazzle, HNK-1, BioLegend), CD95 (BV650, DX2, BioLegend), and viability dye (e780, 1:100, eBioscience). Samples were acquired on a CytoFLEX S flow cytometer (Beckman Coulter) and analysed using FlowJo v10 software (BD). CD25 expression was determined as the median fluorescence intensity (MFI) on CD4^+^ T cells and CD8^+^ T cells.

### 2.5 In vitro stimulation

Cryopreserved PBMCs were thawed in three separate batches, each comprising an equal number of samples from the different cohorts. Cells were washed, and 150,000 cells/well were either measured directly without stimulation or seeded into a 96-well plate in RPMI-1640 medium supplemented with anti-CD3/CD28 (Dynabeads) and incubated overnight at 37°C with 5% CO_2_ for T-cell stimulation. The expression of T-cell activation markers was measured using an LSRFortessa (BD Biosciences) flow cytometer with the following antibody panel: CD3-AF700, CD4-BV510, CD8-APC, CD25-BV421, CD127-PE-Cy7, CD69 (BV650, FN50, BioLegend), and CD40L (PE, 24-31, BioLegend).

### 2.6 Calculations and statistics

Flow cytometry data were processed using FlowJo software. Statistical analyses were performed using IBM SPSS Statistics v29, GraphPad Prism 10, and R v4.0 [17]. Normality was assessed via Shapiro-Wilk tests; non-parametric data were compared using Mann-Whitney U (two groups) or Kruskal-Wallis with Dunn's post-hoc correction (≥3 groups). Multivariate linear regression was performed in R, with age and gender (sex) as covariates. Four outcomes were examined: CD4^+^ CD25 MFI, CD8^+^ CD25 MFI, CD4^+^ CD25^+^CD127+ frequency, and CD8^+^ CD25^+^CD127+ frequency. Group comparisons were structured around one reference category: the healthy control group (HI vs. Control, MID vs. Control, and LOW vs. Control). Results are reported as standardised β coefficients with 95% confidence intervals and two-tailed p-values; p < 0.05 was considered statistically significant.

### 2.7 Data availability

The datasets generated during and/or analysed in the current study are available from the corresponding author upon reasonable request.

## 3 Results

### 3.1 T cell phenotyping and CD25 expression

**Fig. 1:**
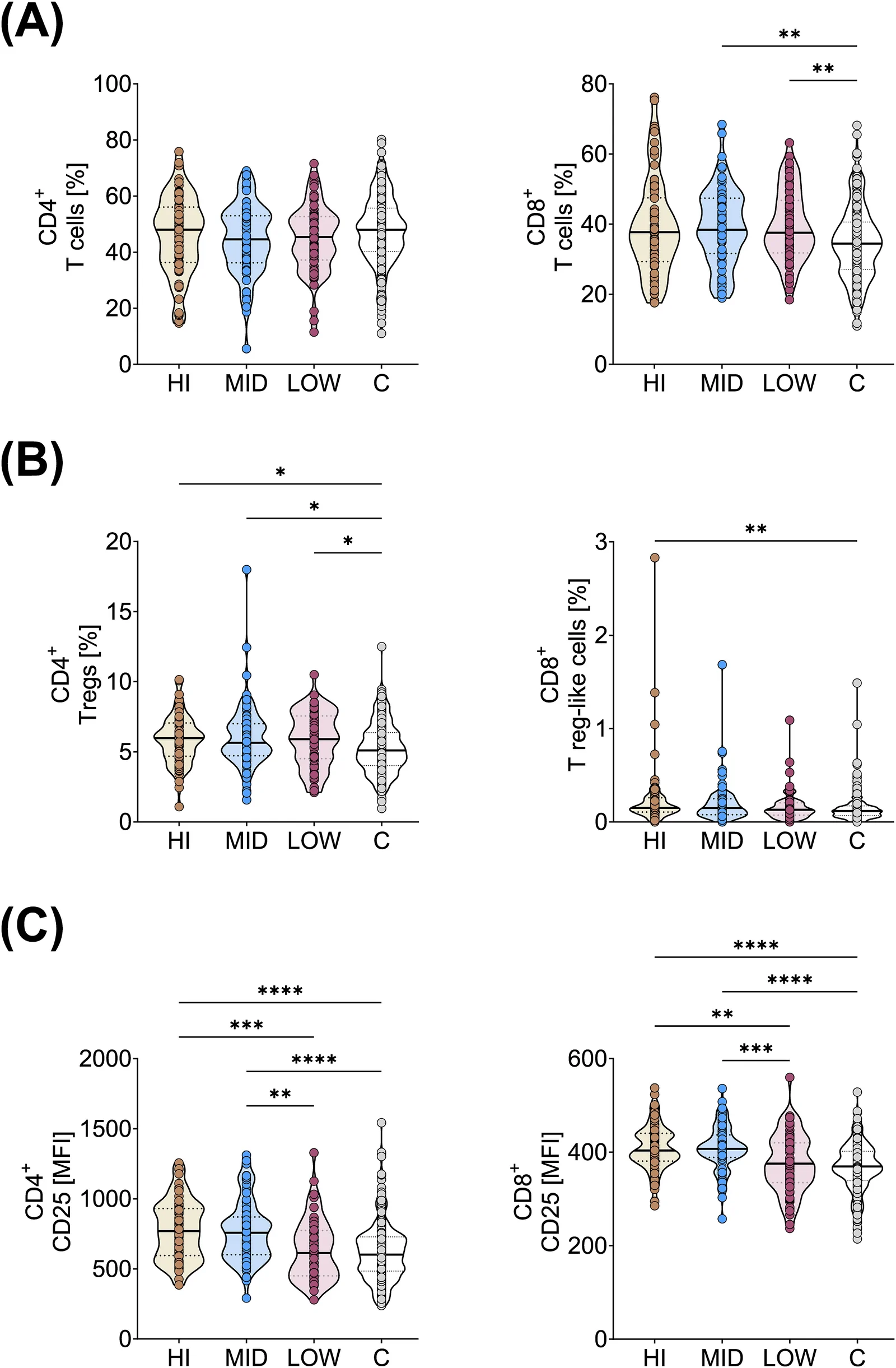
T cell proportions and CD25 expression across clinical cohorts. (a) Flow cytometric quantification of peripheral CD4 and CD8 T cell subsets in different C-peptide groups compared to healthy controls (C). (b) Frequencies of CD4^+^ and CD8^+^ regulatory T cells (Tregs), defined as CD25^+^CD127^−^. (c) Surface expression (Median Fluorescence Intensity, MFI) of CD25 on total CD4^+^ and CD8^+^ T cells. Data are stratified by C-peptide levels: High (HI), Mid (MID), and Low (LOW), alongside controls. Data are presented as violin plots with medians and quartiles. Statistical significance was determined using Kruskal-Wallis tests followed by Dunn's post-hoc correction (∗: p < 0.05, ∗∗: p < 0.01, ∗∗∗: p < 0.001, ∗∗∗∗: p < 0.0001).

T cell immunophenotyping was performed in Ghanaian individuals who were clinically diagnosed with T1D and had been undergoing long-term insulin treatment. These individuals were categorised into groups according to their C-peptide levels: high (HI) and intermediate (MID) levels, which suggest atypical diabetes presentations, and low (LOW) levels, which are indicative of classical T1D, as described previously [2]. Age- and sex-matched healthy controls were included in the study. Detailed cohort characteristics are provided in Table 1, and the gating strategy is depicted in Suppl. Fig. 1. A comprehensive analysis of T cell populations across various C-peptide groups and healthy controls indicated no significant differences in the CD4 T cell proportions. However, there was an observed increase in CD8 T cells among individuals in the C-pep MID and LOW groups compared to controls (Fig. 1A). Notably, a significant increase in CD4 Tregs (identified based on CD25 and CD127 expression) was observed across all diabetes subgroups compared to healthy controls (Fig. 1B). In CD8 T cells, an increase in T regulatory-like CD8 cells was observed exclusively in individuals from the C-pep HI group (Fig. 1B). This was accompanied by a marked elevation in general CD25 MFI expression on both CD4 and CD8 T cells, with the most pronounced differences observed in individuals classified within the C-peptide MID and HI groups compared to both the C-peptide LOW and control groups (Fig. 1C). The findings revealed variations in CD25 expression linked to T cells among individuals diagnosed with T1D in Ghana, with the most significant increases observed in the C-peptide HI and MID subgroups.

### 3.2 Correlation of CD25 expression with clinical parameters

**Fig. 2:**
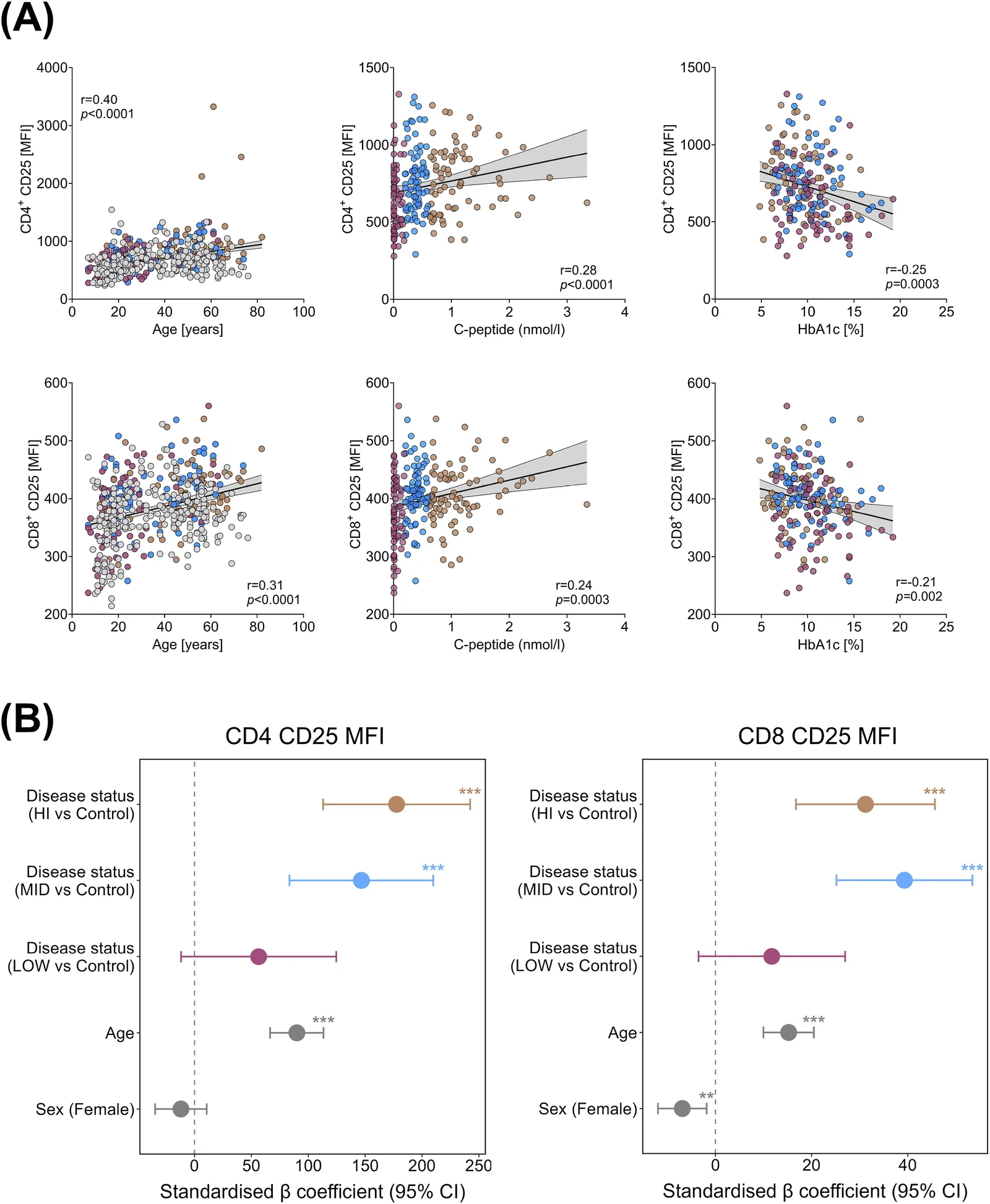
Correlation of CD25 expression with clinical and metabolic parameters. (a) Spearman correlation analyses of CD25 MFI on CD4^+^ and CD8^+^ T cells with age, C-peptide levels, and HbA1c. (b) Forest plots displaying β-coefficients with 95% confidence intervals from multivariable linear regression models predicting CD25 MFI on CD4^+^ and CD8^+^ T cells. Models were adjusted for sex, age, and disease status/C-peptide group**.**

Further investigation of the clinical relevance of CD25 expression revealed significant correlations with key demographic and metabolic parameters. CD25 expression on both CD4 and CD8 T cells showed a positive correlation with age (r = 0.4 and p < 0.0001 for CD4 and r = 0.31 and p < 0.0001 for CD8) as well as with C-peptide levels (r = 0.28 and p < 0.0001 for CD4 and r = 0.24 and p =0.0003 for CD8), indicating that older individuals and those with higher residual beta-cell function tended to exhibit higher CD25 expression (Fig. 2A). Conversely, CD25 expression was negatively correlated with HbA1c levels (r = −0.25 and p = 0.0003 for CD4 and r = −0.21 and p = 0.002 for CD8, Fig. 2A). To allow for the simultaneous assessment of multiple correlated predictors and identification of independent contributions, we performed multivariate linear regression analyses that included age, sex, and C-peptide group assignments. The results revealed that both age and assignment to the MID and HI C-peptide groups independently predicted elevated CD25 expression levels on both CD4 and CD8 T cells (Fig. 2B). The findings underscored the intricate association between clinical conditions and demographic factors in influencing CD25 expression patterns in diabetes, with particular emphasis on the impact of age and disease status.

### 3.3 IL2RA genotyping and association with CD25 expression

**Fig. 3:**
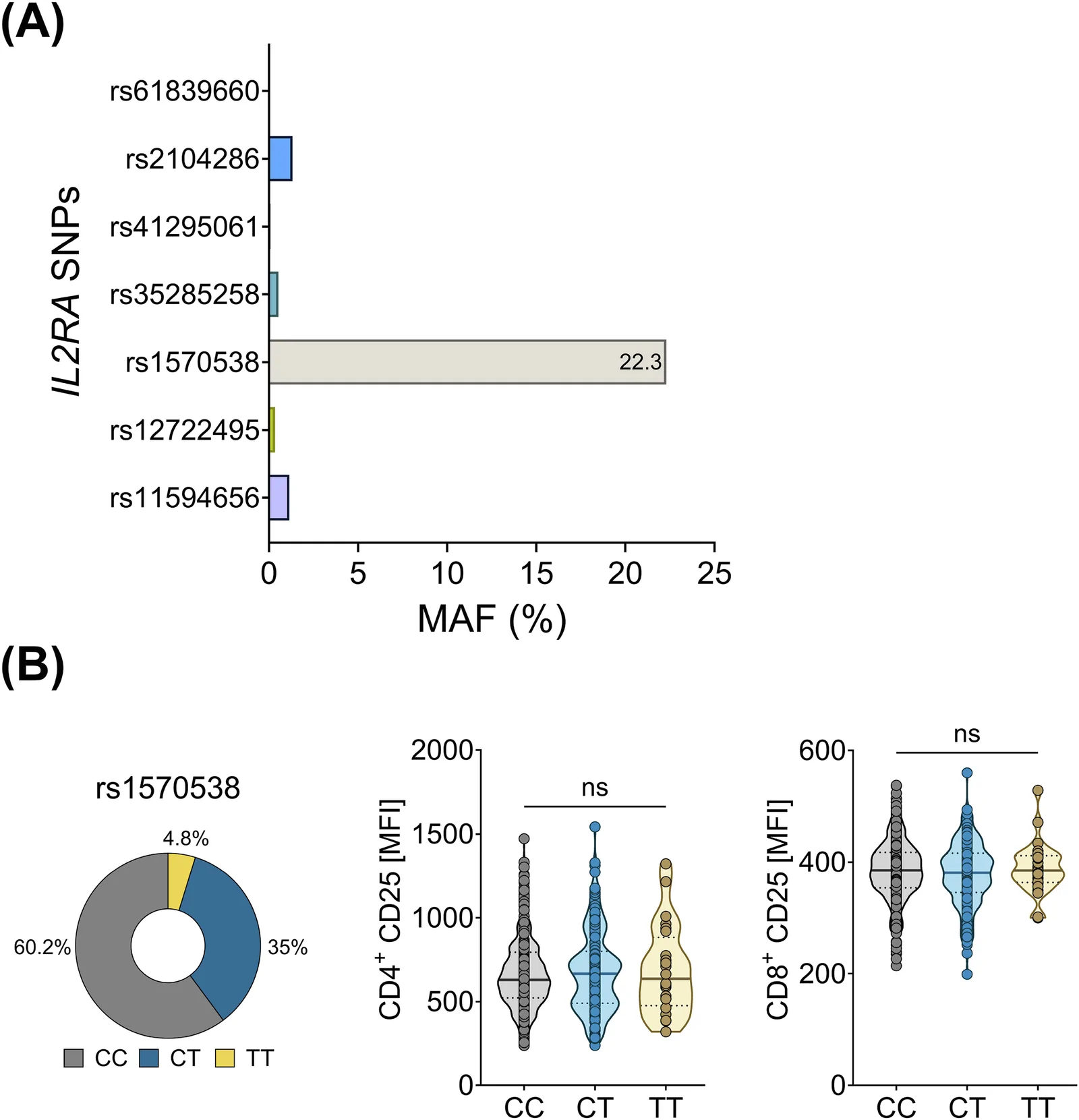
Genetic architecture of the *IL2RA* locus in the Ghanaian cohort. (a) Minor allele frequencies (MAF) of seven selected *IL2RA* single nucleotide polymorphisms (SNPs). (b) Genotype distribution for rs1570538, shown as a doughnut chart, and its association with CD25 expression (MFI) on CD4^+^ and CD8^+^ T cells. Differences across genotypes were assessed using Kruskal-Wallis tests with Dunn's post hoc correction.

To explore the potential influence of genetic factors on CD25 expression, genotyping of IL2RA SNPs previously implicated in modulating IL2R expression was performed in the Ghanaian cohort. Compared to European populations, this cohort exhibited low genetic variability at these loci (Fig. 3A). Notably, SNP rs1570538, the only SNP with higher genetic diversity (minor allele frequency of 22.3%), showed no significant association with CD25 expression on either CD4 or CD8 T cells in this population (Fig. 3B). These findings suggested that genetic factors, which have been shown to influence IL2RA expression in European cohorts, may not have the same impact on the Ghanaian population studied, highlighting the importance of population-specific genetic backgrounds in immunogenetic research.

### 3.4 CD25/CD127 double-expressing cells in diabetes subcohorts

**Fig. 4:**
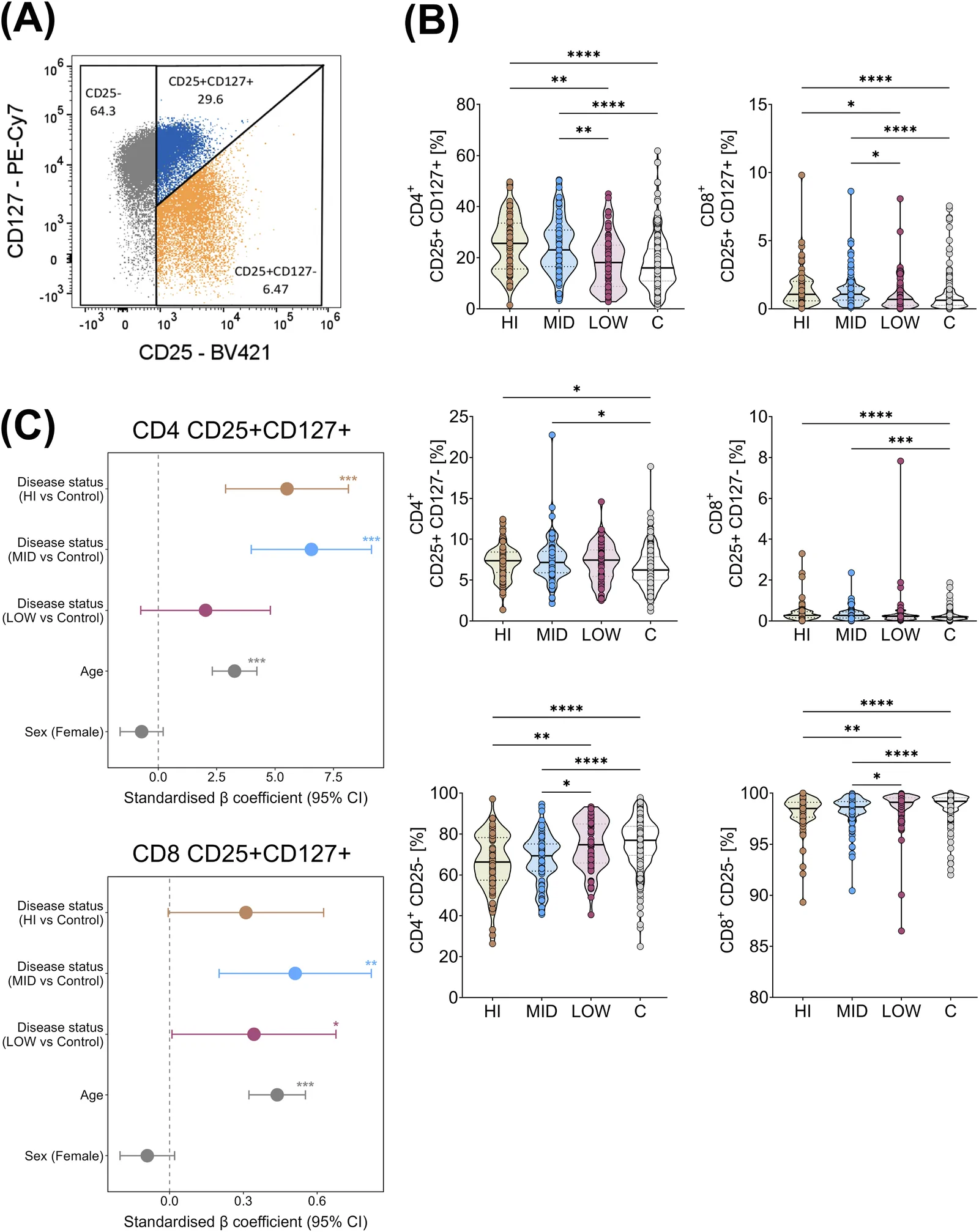
Identification and distribution of CD25^+^CD127^+^ conventional T cells. (a) Representative flow cytometry dot plot illustrating the hierarchical gating strategy to distinguish CD25^−^ (negative), CD25^+^CD127^+^ (double-positive), and CD25^+^CD127^−^ (single-positive) T cell populations. (b) Proportions of these subsets within the CD4^+^ and CD8^+^ T cell compartments stratified by study group. (c) Standardized β-coefficients (95% CI) from multivariable linear regression identifying independent predictors of CD25^+^CD127^+^ subset frequency. Statistical comparisons were performed via Kruskal-Wallis with Dunn's post-hoc correction (∗: p < 0.05, ∗∗: p < 0.01, ∗∗∗: p < 0.001, ∗∗∗∗: p < 0.0001).

Recognising that CD25 expression is not exclusive to Tregs but is also found on activated conventional T cells, this study further dissected the proportions of CD25^+^ single-positive and CD25/CD127 double-positive cells within the T cell compartment. This distinction is critical because CD127 expression differentiates conventional T cells with high CD25 expression (CD25/CD127 double-positive cells) from Tregs, which are typically CD127 low. The gating procedure is depicted in Fig. 4A. The analyses revealed the most pronounced differences between groups for CD25/CD127 double-positive CD4 and CD8 T cells, which were significantly more common in the C-peptide MID and HI groups than in the LOW and control groups (Fig. 4B, upper panel). CD25 single-positive CD4 and CD8 T cells were also increased in the C-peptide HI and MID cohorts, but only compared to controls (Fig. 4B, middle graph). CD25-negative cells were lower in the C-peptide MID and HI groups than in the C-peptide LOW and control groups (Fig. 4B, bottom graph). Multivariate regression analysis, which included parameters such as disease status, age, and sex, demonstrated that assignment to the C-peptide MID and HI groups, compared to the control group, was independently associated with elevated frequencies of CD25/CD127 double-positive CD4^+^ T cells (Fig. 4C). For CD25/CD127 double-positive CD8 T cells, both assignment to the C-peptide LOW and MID cohorts, in contrast to the control group, and age were independently associated (Fig. 4C). These findings indicated a selective expansion of the subset of CD25/CD127 double-positive conventional T cells across the T1D cohorts in Ghana. Furthermore, they emphasised the significance of age in influencing CD25 expression levels on T cells.

### 3.5 Further characterization of CD25 CD127 double-expressing cells

**Fig. 5:**
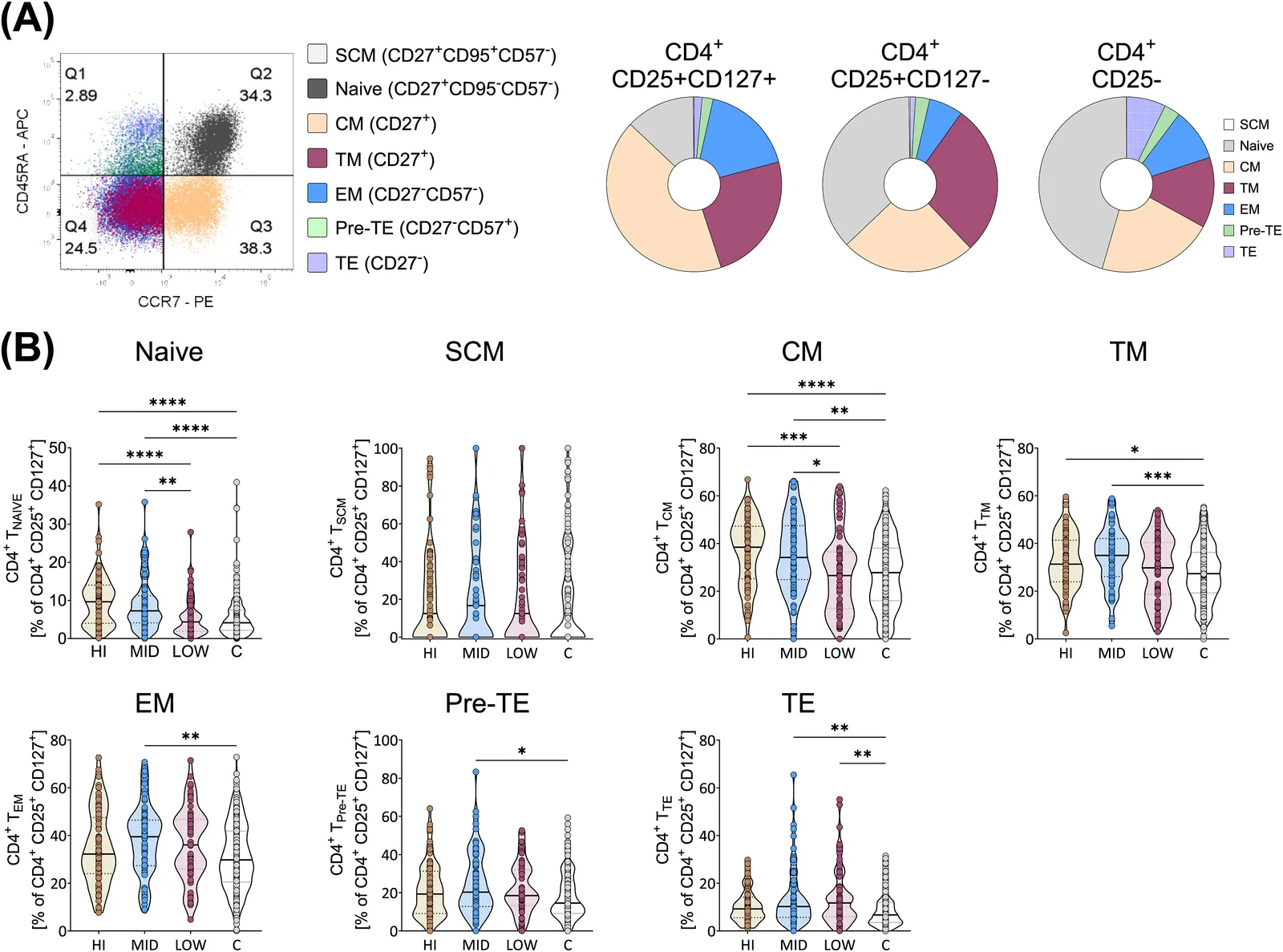
Memory subset distribution within CD25^+^CD127+ conventional CD4^+^ T cells. (a) Representative flow cytometry dot plot illustrating hierarchical gating strategy for seven T cell maturation stages: Naïve (CD45RA^+^CCR7^+^; CD27^+^CD95^−^CD57^−^), Stem Cell Memory (SCM, CD45RA^+^CCR7^+^; CD27^+^CD95^+^CD57^−^), Central Memory (CM, CD45RA^−^CCR7^+^; CD27^+^), Transitional Memory (TM, CD45RA^−^CCR7^-^; CD27^+^), Effector Memory (EM, CD45RA^−^CCR7^-^; CD27^−^), Pre-Terminal Effector (Pre-TE, CD45RA^+^CCR7^-^; CD27^+^), and Terminal Effector (TE, CD45RA^+^CCR7^-^; CD27^−^) within the CD25/CD127 compartments. (b) Frequencies of CD25^+^CD127+ cells across identified memory subsets stratified by study group. Data are shown as violin plots with medians. Statistical analysis via Kruskal-Wallis with Dunn's post-hoc correction (∗: p < 0.05, ∗∗: p < 0.01, ∗∗∗: p < 0.001, ∗∗∗∗: p < 0.0001).

To achieve a more comprehensive understanding of the phenotypic characteristics of expanded CD25/CD127 double-positive T cells, subsequent analyses focused on their distribution among the memory T cell subsets. Within the CD25/CD127 double-positive CD4 T cell population, we evaluated the proportions of stem cell memory cells (SCM), naïve cells, central memory (CM), transitional memory (TM), effector memory (EM), pre-terminal effector (Pre-TE), and terminal effector cells (TE), and compared these proportions to those observed in CD25 single-positive and CD25 negative CD4 T cells (Fig. 5A and B). Notably, the CM subset, and to a lesser extent the EM subset, was larger in the CD25/CD127 double-positive compartment than in the single and CD25^−^negative compartments (Fig. 5A and Supplementary Fig. 2). In contrast, only few naïve, Pre-TE, and TE cells were found among the CD25/CD127 double-positives (Fig. 5A and Supplementary Fig. 2). Having established that CM cells dominate among the double-positive cells, we investigated whether CM cells or other subsets exhibited marked cohort differences in the proportion of double-positive cells. To this end, we compared the proportion of CD25/CD127 double-positive cells in the memory subsets across the cohorts. Indeed, the pattern of an increased proportion of CD25/CD127 double-positive cells in the C-peptide HI and MID groups was found primarily in CM cells, but also in naïve and TM cells (Fig. 5B). The findings indicated that the variation in CD25 expression on CD4 T cells observed across the diabetes cohorts is linked to distinct stages of T cell maturation.

### 3.6 CD25 regulation after activation

**Fig. 6:**
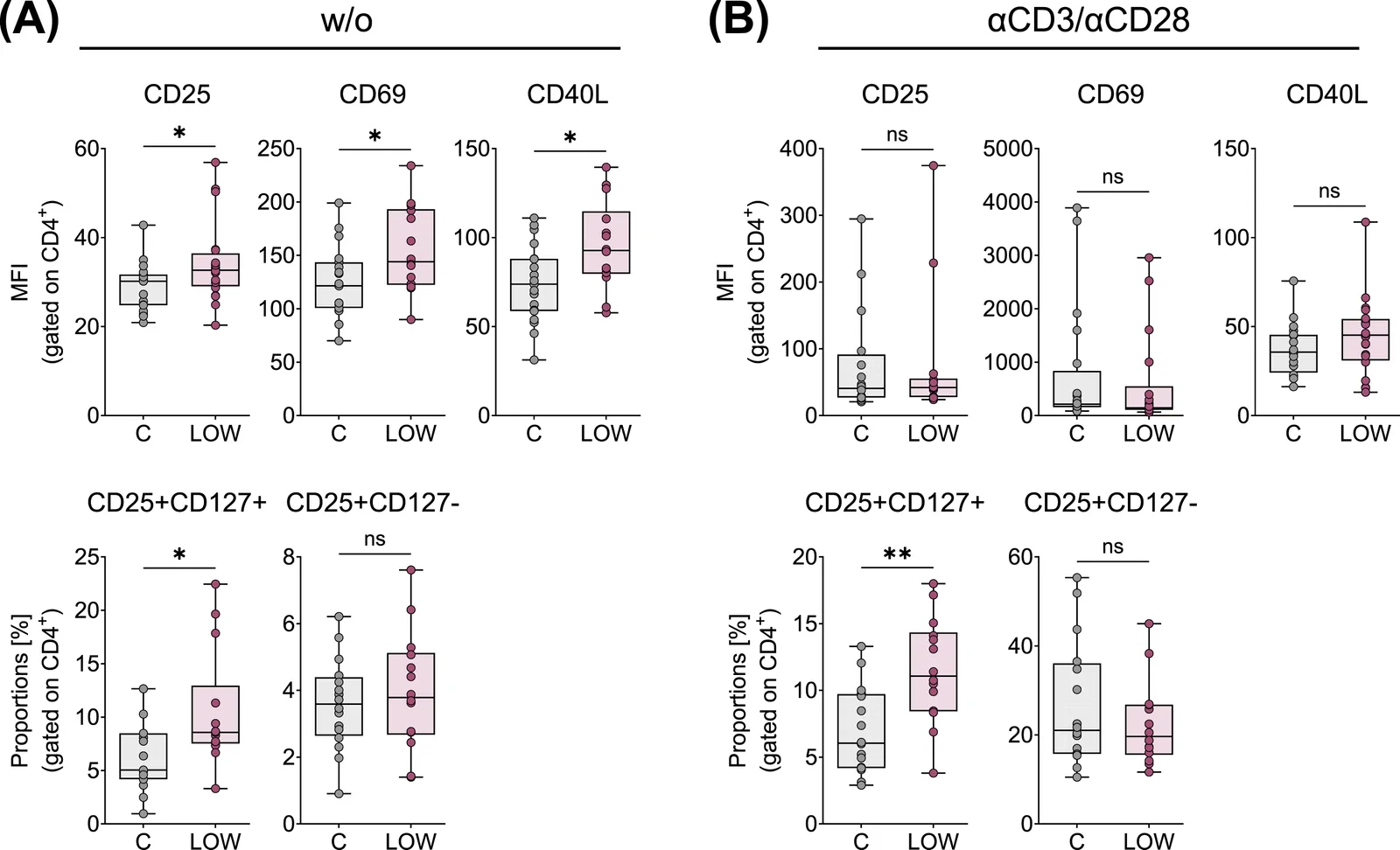
CD25 regulation and T cell activation after in vitro stimulation. (a) Ex vivo expression (MFI) of CD25, CD69, and CD40L and frequencies of CD25^+^CD127^+^ and CD25^+^CD127^-^ on T cells in an age- and sex-comparable subcohort of LOW C-peptide individuals and healthy controls. (b) Activation marker expression and CD25/CD127 frequencies following in vitro stimulation with anti-CD3/CD28. Data are presented as box plots showing individual values and medians. Statistical significance was assessed using Mann-Whitney U tests (ns = not significant, ∗ p < 0.05, ∗∗ p < 0.01).

Initial findings showed that changes in CD25 expression were most noticeable in the C-peptide HI and MID groups. However, the results for the C-peptide LOW T1D group were less clear. To address this and reduce the possible effects of age, a subcohort of 13 individuals with LOW C-peptide levels was chosen and matched with a control group of 20 healthy individuals, ensuring that they were similar in age and sex (Suppl. Table 1). These subcohorts were used to examine CD25 expression. Since CD25 on CD25/CD127 double-positive T cells likely indicates an increased activation status, additional activation markers on T cells were included, both ex vivo and after in vitro T cell receptor stimulation. Directly ex vivo, CD25 expression on CD4 T cells was elevated in C-peptide LOW individuals compared to controls (Fig. 6A). Similarly, increased expression of the activation markers CD69 and CD40L was observed (Fig. 6A). When differentiating into CD25/CD127 double-positive and CD25 single-positive CD4 T cells, we found that only the double-positive cells were increased in the C-peptide LOW group (Fig. 6A). Following T cell activation for 16 h, no group differences were observed in general CD25, CD69, and CD40L expression on CD4 T cells (Fig. 6B). However, the CD25/CD127 double-positive compartment remained significantly elevated in T1D with LOW C-peptide levels after activation (Fig. 6B). In CD8 T cells, no differences in the MFI of CD25, CD69, and CD40L were observed between participants with T1D and control subjects, both ex vivo and following activation (Suppl Fig. 3A and B). However, the CD25/CD127 double-positive compartment was elevated in individuals with low C-peptide levels under both ex vivo and stimulated conditions (Suppl Fig. 3A and B). These results underscored the presence of an activated T cell phenotype also in T1D exhibiting low C-peptide levels, with a specific emphasis on the role of CD25/CD127 double-positive cells.

## 4 Discussion

Immunophenotyping of cohorts with T1D and atypical diabetes demonstrated a significant increase in T cell-associated CD25 expression levels. This increase was mainly found in conventional T cells co-expressing CD25 and CD127 and was observed in individuals with low, mid, and high C-peptide. Notably, these CD25/CD127 double-positive cells predominantly exhibited a central memory phenotype and were elevated both ex vivo and following T cell stimulation, indicating an overall heightened activation status of CD4^+^ T cells across diabetes subtypes.

When discussing the significance of CD25 expression in autoimmune diseases and T1D, the focus is often on CD25 expression on Tregs. We identified Tregs by applying the standard practice of using CD25 and CD127 for gating [18] and observed a moderate increase in the proportion of CD4 Tregs across all diabetes subcohorts with HI, MID, and LOW C-peptide levels compared with controls. Tregs have been extensively studied in T1D, revealing a functional defect despite often normal frequencies, however, lower and higher Treg numbers have also been described [12,[19], [20], [21]]. The role of Tregs in type 2 diabetes and other forms of diabetes remains unclear. Within the framework of chronic low-grade inflammation induced by metabolic dysfunction, obesity, and insulin resistance in type 2 diabetes, some alterations in Treg phenotype and function have been documented. Both decreased and increased numbers of CD4 Tregs have been reported [22,23], with predominant observations of diminished regulatory function [11] and disrupted Treg/effector T cell balance [24].

Alongside the observed increase in Treg numbers, we identified a notable elevation in CD25 expression on CD4 and CD8 T cells among individuals with HI, MID, and - when controlling for the age effect - also LOW C-peptide levels. This finding contrasts with earlier studies in T1D, which documented a reduction in CD25 expression that was associated with diminished IL-2 responsiveness [7,25]. However, these studies reported changes in CD25 expression in Tregs, the T cell population that constitutively expresses CD25, and these modifications were linked to a reduced suppressive capacity. It is important to note that CD25 serves not only as a marker on Tregs but also as an activation marker on conventional T cells. Upon T cell receptor engagement and costimulation, conventional naïve and memory T cells rapidly increase CD25 expression, which enables them to respond to IL-2, undergo differentiation, and promote survival and effector function [26]. In T1D, it has been reported that conventional T cells, which are islet-specific, exhibit elevated levels of CD25, making them particularly sensitive to IL-2 [27]. In general, cells with high CD25 expression sequester IL-2 and limit its availability to effector cells, thereby suppressing the immune response [28]. On the other hand, when cells have impaired IL-2 signalling, they are less effective at capturing IL-2, rendering more IL-2 available for other cells [29].

Our study identified an increase in CD25/CD127 double-positive T cells across the C-peptide MID, HI, and LOW groups. The role of CD25/CD127 double-positive cells remains a subject of debate. On the one hand, this subset has been described as having anti-inflammatory and regulatory functions. In T1D, Narsale et al. reported that CD25/CD127 double-positive CD4 T cells exhibit an anti-inflammatory Th2-type bias, with individuals showing the highest frequency of these cells at T1D diagnosis being significantly more likely to maintain β-cell function [30,31]. In the context of acute pancreatitis, CD25/CD127 double-positive cells have been associated with protection against multiple organ failure, suggesting an anti-inflammatory role in mitigating excessive systemic inflammation [32]. Di Caro et al. demonstrated that CD25/CD127 double-positive cells were comparably suppressive to CD25^+^ CD127- Tregs in in vitro suppression assays, although their focus was on double-positive cells also expressing FoxP3, which constitutes only a minor subset [33,34]. Conversely, other studies have documented that CD25/CD127 double-positive cells exhibited proinflammatory effector functions during immune activation or disease. In autoimmune hepatitis and autoimmune sclerosing cholangitis, CD127/CD25 double-positive cells lacked suppressive functions, displayed a mixed Th1/Th17 phenotype, and promoted inflammation [35]. The implications of increased proportions of CD25/CD127 double-positive cells appear to depend on several factors, including the FoxP3 expression status, activation state, and cytokine environment. Coexpression of FoxP3 in double-positive cells and the presence of pro-inflammatory cytokines have been described as contributing to effector differentiation, while IL-7 stimulation could induce a transition to classical CD25^+^ CD127- Tregs [[33], [34], [35]]. Taken together, CD25/CD127 double-positive cells represent a heterogeneous population with context-dependent function. Given the increased proportions observed in all of our diabetes cohorts, irrespective of diabetes classification, a direct association with autoimmunity seems unlikely. Instead, upregulation may be linked to the inflammatory milieu present in diabetes, either as a consequence or as a compensatory mechanism.

The observed associations between CD25 expression and clinical parameters highlight age as a critical factor influencing CD25 levels on T cells. Accordingly, differences in CD25 expression between healthy controls and individuals with low C-peptide levels were only apparent in age-matched cohorts. This underscores the fact that age-related changes in immune activation can confound direct comparisons of CD25 expression across groups. The positive correlation between CD25 expression and age suggests that immune system maturation or senescence may modulate IL-2 receptor expression, which potentially impacts both regulatory and conventional T cell populations. Other studies that reported a positive association between age and CD25 expression have primarily focused on Tregs [[36], [37], [38], [39]]. Future studies investigating CD25 as a biomarker in diabetes should carefully control for age to avoid misinterpretation of immunophenotypic differences.

This study had some limitations. One limitation is the absence of functional data assessing IL-2 responsiveness, such as pSTAT5 levels following IL-2 stimulation. Moreover, the FoxP3 expression in the CD25/CD127 double-positive cell population was not investigated. This restricts the ability to fully characterise the functional status and regulatory potential of these cells in the inflammatory context of diabetes.

Taken together, this study integrated cellular phenotyping, clinical correlations, and potentially relevant genetic factors to improve the understanding of immune heterogeneity in diabetes within an underrepresented African population. The results emphasise the importance of considering both regulatory and conventional T cell subsets when investigating the immunopathology of diabetes. They also highlight the involvement of CD25-expressing T cell subsets in the immunopathology of diabetes, supporting the rationale for targeting CD25 in immunomodulatory therapies currently under development for T1D and other autoimmune disorders.

## CRediT authorship contribution statement

**Wilfred Aniagyei:** Writing – original draft, Visualization, Validation, Software, Methodology, Investigation, Formal analysis, Data curation. **Sophie L. Womelsdorf:** Writing – review & editing, Investigation, Formal analysis. **Sarah Bittner:** Writing – review & editing, Investigation, Formal analysis. **Sumaya Mohayideen:** Writing – review & editing, Investigation. **Osei Sarfo-Kantanka:** Writing – review & editing, Resources, Conceptualization. **Ernest Adankwah:** Writing – review & editing, Supervision. **Dorcas O. Owusu:** Writing – review & editing, Supervision. **Hillary Y.B. Nafu:** Writing – review & editing, Investigation. **Monika M. Vivekanandan:** Writing – review & editing, Investigation. **Augustine Yeboah:** Writing – review & editing, Investigation. **Joseph F. Arthur:** Writing – review & editing, Investigation. **Hubert S. Ahor:** Writing – review & editing, Investigation. **Vera Balz:** Writing – review & editing, Investigation, Formal analysis. **Shadrack Osei Asibey:** Writing – review & editing, Resources, Investigation. **Agnes Owusu Boateng:** Writing – review & editing, Resources, Investigation. **Elisabeth Owusu:** Writing – review & editing, Resources, Investigation. **Ertan Mayatepek:** Writing – review & editing, Conceptualization. **Marc Jacobsen:** Supervision, Resources, Conceptualization. **Richard O. Phillips:** Writing – review & editing, Supervision, Resources, Conceptualization. **Julia Seyfarth:** Writing – original draft, Visualization, Validation, Supervision, Resources, Project administration, Methodology, Investigation, Funding acquisition, Formal analysis, Data curation, Conceptualization.

## Ethics declaration

Written informed consent to take part in the study has been obtained from all participants (older than 14 years) and/or their legal representatives. The privacy rights of participants have been observed.

This study included organ or tissue donors. This study includes human biological material and consent was obtained by donors, or their next of kin or legal representatives, for use in this study and for publication of the article. The samples used in this research were not sourced from executed prisoners or prisoners of conscience.

This study was performed in compliance with relevant laws, regulatory frameworks and guidelines where the research took place. This study was approved by the Ethics Committee Board at KATH. (Approval No. KATH IRB/AP/081/20)

## Funding

This work was supported in part by a grant from the Else Kröner-Fresenius Foundation and Elterninitiative Kinderkrebsklinik e.V. to J. S. The funder was not involved in the study design, the collection, analysis, and interpretation of the data, the writing of the report, or the decision to submit the article for publication.

## Declaration of competing interest

The authors declare the following financial interests/personal relationships which may be considered as potential competing interests: Julia Seyfarth reports financial support was provided by Else Kröner-Fresenius Foundation. Julia Seyfarth reports financial support was provided by Elterninitiative Kinderkrebsklinik e.V. If there are other authors, they declare that they have no known competing financial interests or personal relationships that could have appeared to influence the work reported in this paper.

## Data Availability

Data will be made available on request.
