# Supplementary material for: Immunophenotypic insights into diabetes heterogeneity: The role of CD25-expressing T cells in Ghanaian type 1 diabetes cohorts
Source: J Transl Autoimmun. 2026 Sep 1;13:100400. doi: 10.1016/j.jtauto.2026.100400 (PMC13572010; doi:10.1016/j.jtauto.2026.100400)
Supplement: Supplementary file 1 [file mmc1.pdf]

Supplementary figure 1

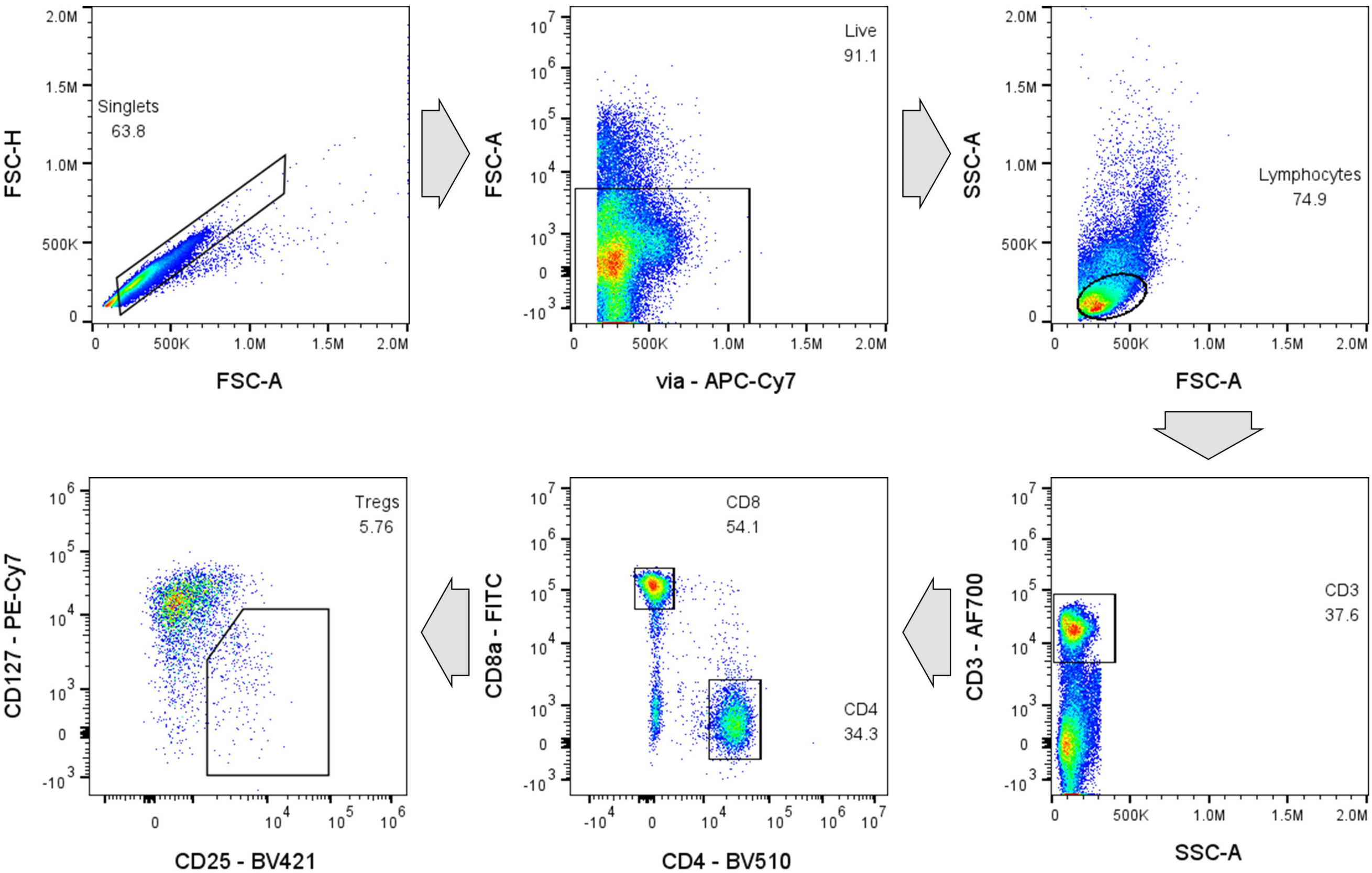

Supplementary Figure 1. Hierarchical gating strategy for the identification of T cell populations. Representative flow cytometry plots illustrating the sequential gating strategy used to isolate target populations. The hierarchy begins with singlet discrimination (FSC-H vs. FSC-A) and viability selection (via-dye vs. FSC-A) to the identification of lymphocytes (FSC-A vs. SSC-A). Subsequent gates define total CD3+ T cells and their bifurcation into CD4+ and CD8+ lineages. The final panel depicts the gating of regulatory T cells (Tregs) based on CD25 and CD127 expression levels.
