# Supplementary material for: Immunophenotypic insights into diabetes heterogeneity: The role of CD25-expressing T cells in Ghanaian type 1 diabetes cohorts
Source: J Transl Autoimmun. 2026 Sep 1;13:100400. doi: 10.1016/j.jtauto.2026.100400 (PMC13572010; doi:10.1016/j.jtauto.2026.100400)
Supplement: Supplementary file 2 [file mmc2.pdf]

Supplementary figure 2

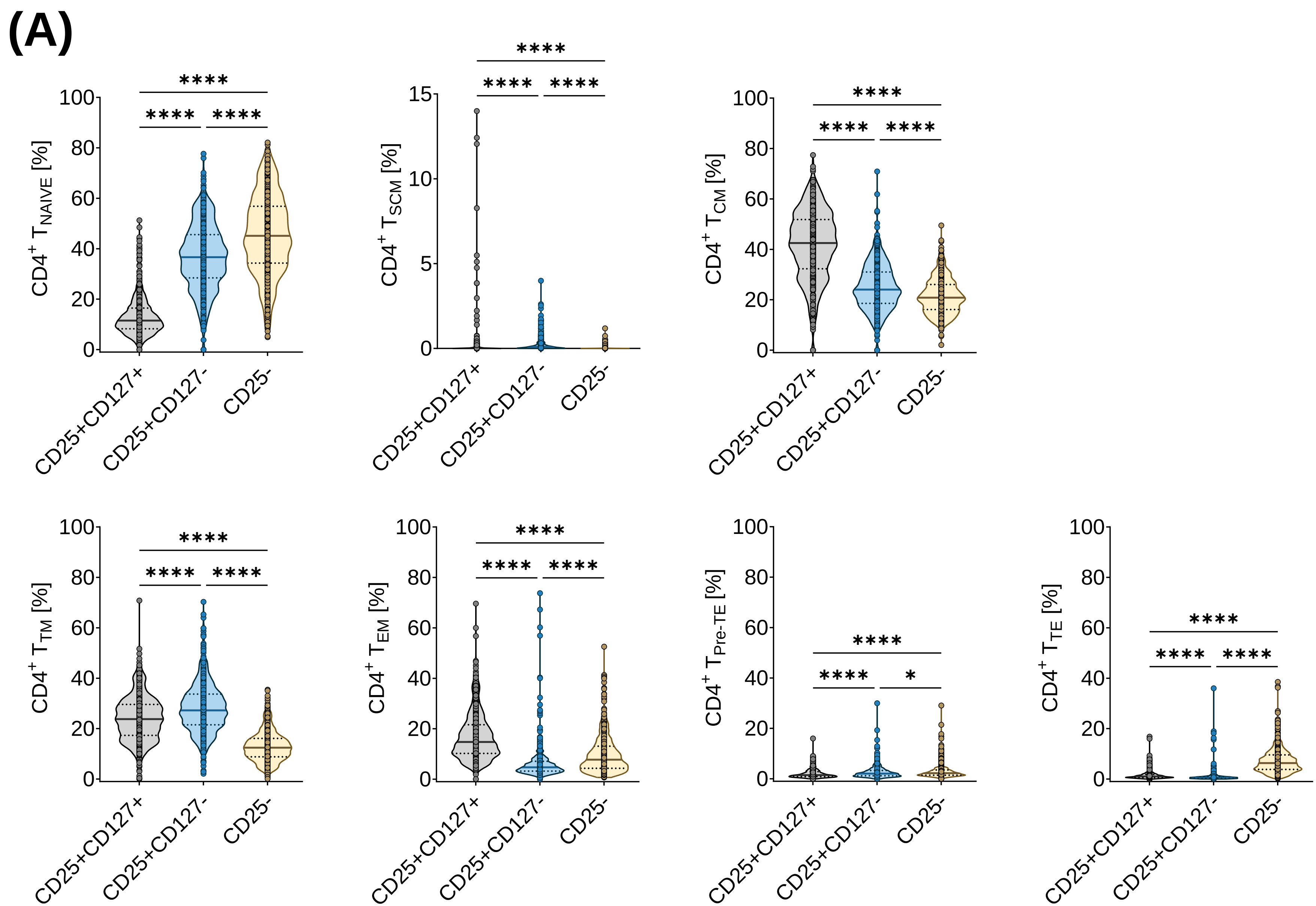

Supplementary Figure 2. Comparison of memory subset distributions across CD25 and CD127 expression profiles. (A) Frequencies of seven maturation stages within the CD4+ T cell compartment. Data are stratified into three distinct populations based on surface markers: $CD25^+CD127^+$, $CD25^+CD127^-$ and $CD25^-$. Data are presented as violin plots with medians and quartiles. Statistical significance across the three expression profiles was determined using Kruskal-Wallis tests followed by Dunns's post-hoc correction.
