# Supplementary material for: Immunophenotypic insights into diabetes heterogeneity: The role of CD25-expressing T cells in Ghanaian type 1 diabetes cohorts
Source: J Transl Autoimmun. 2026 Sep 1;13:100400. doi: 10.1016/j.jtauto.2026.100400 (PMC13572010; doi:10.1016/j.jtauto.2026.100400)
Supplement: Supplementary file 3 [file mmc3.pdf]

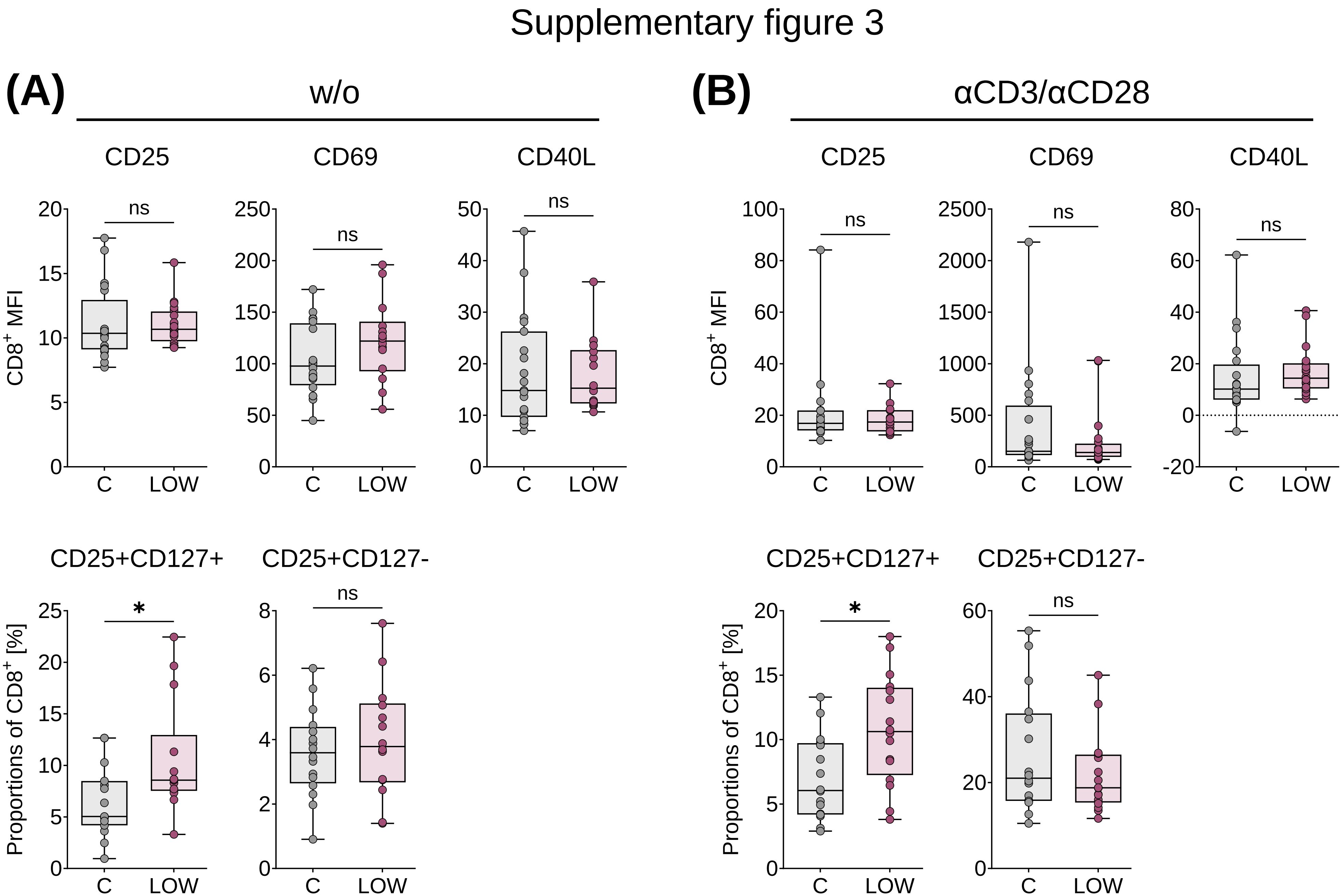

Supplementary Figure 3. CD8+ T cell activation and CD25 regulation. (a) Ex vivo expression (MFI) of CD25, CD69, and CD40L and frequencies of $CD25^+CD127^+$ and $CD25^+CD127^-$ on T cells in an age- and sex-comparable subcohort of LOW C-peptide individuals and healthy controls. (b) Activation marker expression and CD25/CD127 frequencies following in vitro stimulation with anti-CD3/CD28. Data are presented as box plots showing individual values and medians. Statistical significance was assessed using Mann-Whitney U tests.
