# Supplementary material for: Immunophenotypic insights into diabetes heterogeneity: The role of CD25-expressing T cells in Ghanaian type 1 diabetes cohorts
Source: J Transl Autoimmun. 2026 Sep 1;13:100400. doi: 10.1016/j.jtauto.2026.100400 (PMC13572010; doi:10.1016/j.jtauto.2026.100400)
Supplement: Supplementary file 4 [file mmc4.docx]

Supplementary Table 1. Characteristics of the *in vitro* cohort

|  | Control | LOW | *p-value* |
| --- | --- | --- | --- |
| Number, *n* | 20 | 14 |  |
| Female/male (*% females*) | 8/12 | 4/10 | 0.72 |
| Age, years | 28.5 | 40 | 0.09 |
| BMI (kg/m^2^) | 23.67 | 25.85 | 0.51 |

The p-values indicate the statistical significance of differences between these two groups, determined using the non-parametric Mann-Whitney U test for continuos variables and chi-square test for categorical variables.
